# Dental patients’ tinnitus profile: prevalence, types, and associated factors with oral and maxillofacial diseases

**DOI:** 10.2340/aos.v83.40572

**Published:** 2024-04-29

**Authors:** Berkan Altay, Elif Çoban, Melike Yurttaş, Özlem Arık, Arif Türkoğlu

**Affiliations:** a Department of Oral and Maxillofacial Surgery, Kırıkkale University, Kırıkkale, Turkey; b Department of Oral and Maxillofacial Radiology, Kütahya Health Sciences University, Kütahya, Turkey; c Department of Biostatistics, Kütahya Health Sciences University, Kütahya, Turkey

**Keywords:** Dentistry, maxillofacial diseases, prevalence, tinnitus

## Abstract

**Introduction:**

Maxillofacial diseases may pose a risk factor for the onset of tinnitus, and may influence the severity of its symptoms. The objective of this study was to investigate the prevalence of tinnitus among patients routinely visiting the Faculty of Dentistry and to assess the relationship between tinnitus and maxillofacial diseases.

**Materials and Methods:**

This was a prospective cross-sectional study conducted on 3,626 patients. Demographic data, information on tinnitus symptoms, temporomandibular disorder (TMD) presence, the existence of trigger points in masticatory muscles, toothache, and bruxism were evaluated.

**Results:**

Tinnitus was detected in 385 patients, resulting in a prevalence rate of 10.61%. Of the patients, 38.4% were male and 61.6% were female, and the mean age was 42.66 ± 16.34 years. Tinnitus was categorised as normal in 47.8% of the patients and pathological in 52.2% of the patients. Bruxism was identified in 65.5% of the patients, toothache in 42.9%, TMD in 33.8%, and masticatory trigger points in 27.0% of the patients. A tendency towards tinnitus provoked by toothache was observed in 5.9% of the patients. The presence of pathological tinnitus was found to increase the risk by 1.839 times for toothache and 1.456 times for bruxism.

**Conclusion:**

There may be an association between oral and maxillofacial diseases and tinnitus, especially bruxism and toothache. Therefore, the evaluation of these conditions may be a routine part of tinnitus management.

## Introduction

Tinnitus is defined as the perception of sounds in the ears or within the head without any acoustic stimulus [1]. Individuals often describe it as ringing, buzzing, humming, or even a steam-like sound [2]. Tinnitus can be categorised as either normal or pathological, depending on the duration of the perceived sound. Normal tinnitus is characterised by episodes lasting less than 5 min without associated hearing loss and resolving in less than a week [3]. Pathological tinnitus, on the other hand, persists for more than 5 min and extends beyond a week, with an unclear underlying mechanism [4]. Tinnitus is further classified into objective tinnitus, where the sound can be heard not only by the patient but also by others, and subjective tinnitus, in which only the patient can perceive the sound [5].

The prevalence of tinnitus among adults varies globally, ranging from approximately 4.1% to 37.2% [6]. Its exact aetiology remains incompletely understood, and may be associated with various risk factors. Maxillofacial diseases are considered potential risk factors for tinnitus development and could impact the severity of its symptoms [7]. Middle ear muscles share a common embryological and functional origin with jaw and facial muscles [8]. Therefore, symptoms referred to the ear can originate from the stomatognathic region. The frequency of tinnitus increases in conditions such as temporomandibular disorder (TMD), sleep bruxism, and myofascial pain [9]. Possible causes of tinnitus in patients with TMD include displacement of the mandibular condyles posteriorly due to changes in molar teeth, resulting in compression of auditory structures; changes in the mobility of the malleus in the middle ear and alteration in tension of the tympanic membrane; excessive mechanical pressure on the discomalleolar ligament or compression on the auriculotemporal nerve; increased tension in jaw muscles leading to excessive loading on adjacent tissues of the temporomandibular joint (TMJ); contraction of the tensor veli palatini and tympanic muscles due to abnormal use of the chewing system, as both muscles share the same origin with jaw muscles and are innervated by the trigeminal nerve, resulting in increased tension of the tympanic membrane and consequently otological symptoms [10–14]. Additionally, it has been reported that the prevalence of tinnitus is higher in patients with TMD and concomitant tooth pain compared to TMD alone [15].

Temporomandibular disorder patients may experience symptoms such as headaches, otalgia, tinnitus, dizziness, hearing loss, sleep disorders, and chronic fatigue [16–19]. Chronic stressors and mental disorders are recognized as potential contributors to the development of bruxism and TMD [20]. However, the precise mechanisms driving this relationship remain incompletely understood. Nonetheless, it is acknowledged that mental health conditions can influence the onset, progression, and response to treatment of TMD [21]. Factors such as sleep habits have also been identified to be associated with bruxism and TMD symptoms in the paediatric population [22]. Stress can also lead to the emergence of systemic diseases, and as a result, TMD and bruxism may occur. Additionally, the SAPHO (Synovitis, Acne, Pustulosis, Hyperostosis, Osteitis) syndrome, playing a role in the pathogenesis of stress, can lead to ankylosis and pain in the TMJ [23, 24]. Temporomandibular joint involvement can be associated with progressive hearing loss, deafness, and tinnitus [25].

Despite the frequent encounter of maxillofacial diseases in individuals with subjective tinnitus in clinical practice, research in this area remains limited. The hypothesis driving this study is that there exists an association between tinnitus and maxillofacial diseases. The primary objective of this investigation was to ascertain the prevalence of tinnitus in patients routinely seeking care at the Faculty of Dentistry. Additionally, the secondary aim was to assess the relationship between tinnitus and maxillofacial diseases.

## Materials and methods

The study was conducted on 3,626 patients who routinely presented to the Faculty of Dentistry at Kütahya Health Sciences University between January 2022 and January 2023. The study was designed as a prospective cross-sectional study. When patients initially presented to the faculty, surveys related to their symptoms were administered by Melike Yurttaş (MY) and Arif Türkoğlu (AT) before the commencement of treatments. The survey included questions about tinnitus symptoms. Clinical examinations have been conducted by Oral and Maxillofacial Radiology and Oral and Maxillofacial Surgery specialists with a minimum of 5 years of experience. Initial examinations concerning maxillofacial pathologies were conducted by MY, followed by Berkan Altay (BA) before the initiation of all patient treatments. It received approval from the local ethics committee under decision number 2021/16-10, and was conducted in accordance with the principles of the Helsinki Declaration.

### Study design

The study population consisted of patients with tinnitus who routinely presented to the Faculty of Dentistry at the Kütahya Health Sciences University. Patients with subjective tinnitus were included in the study. Patients with objective tinnitus, and those who have been actively treated for tinnitus, TMD, or bruxism currently or in the past, as well as emergency cases, were excluded from the study. The initial examinations for patients in terms of oral and maxillofacial aspects were conducted by an Oral and Maxillofacial Radiology specialist. Demographic data, examination findings, and tinnitus data were recorded. Patients were asked questions regarding the types of tinnitus they experienced. Tinnitus that was not audible to the physician and not associated with muscle myoclonus was considered subjective tinnitus. Tinnitus that occurred without hearing loss, lasted less than 5 min, and resolved in less than 1 week was classified as normal tinnitus, while tinnitus lasting longer than 5 min and persisting for more than 1 week was classified as pathological tinnitus. Patients were asked about the duration, frequency, which ear they felt the tinnitus in, and whether there were any factors that increased/decreased the intensity of their tinnitus. Patients experiencing toothaches were referred to the Department of Restorative Dentistry and Endodontics. Patients with toothaches associated with TMD, bruxism, trigger points in masticatory muscles, and indications for extraction were re-evaluated by an Oral and Maxillofacial Surgery specialist based on these findings. The examination findings were recorded and compared. In cases where the examination findings overlapped with initial examinations, a third independent opinion was sought from an experienced oral and maxillofacial surgeon. The clinical diagnosis of TMD and the examination of physical findings were conducted according to the Research Diagnostic Criteria for Temporomandibular Disorders (RDC/TMD) Axis I. The patients’ symptoms, complaints, and medical/dental histories were recorded. Pain, incisal relationships, jaw opening pattern, jaw opening movements, lateral and protrusive movements, TMJ sounds during opening and closing, TMJ sounds during lateral and protrusive movements, joint locking, pain elicited by palpation of muscles and TMJ, and additional muscle pain elicited by palpation were documented. Temporal, masseter, lateral and medial pterygoid, sternocleidomastoids, and posterior cervical muscles were examined by direct palpation and functional manipulation. Trigger points, reflected pain, muscle asymmetry and hypertrophy were assessed. Teeth were examined for caries, mobility, and abrasions. The clinical examination of the patients determined centric relation and centric occlusion. Occlusion, arch integrity, vertical occlusal dimension, eccentric occlusal contacts, protrusive, laterotrusive and mediotrusive contacts were checked. The type of occlusion was determined in clinical examination. Bruxism was assessed based on clinical examination and patient history. In terms of bruxism, attrition, abrasion, corrosion, and abfraction wear and fractures in teeth and restorations, linea alba, and changes in shape observed at the edges where the tongue contacts the teeth were examined.

### Statistical methods

When calculating the sample size required for the study, the prevalence of tinnitus, that is, the incidence of tinnitus, was taken into account. For 50% prevalence, power analysis was performed using *d* = 0.05 tolerance amount (acceptable error) and 0.05 margin of error (95% confidence level). A minimum power value of 80% was obtained when working with a sample of 385 individuals. GPower 3.1 program was used for power analysis. Descriptive statistics, including measures such as mean, standard deviation, maximum, and minimum, were employed to analyse quantitative data. For categorical or qualitative data, frequency tables presenting frequencies and percentages were utilised. To examine relationships between categorical variables, double or triple cross tables and chi-squared tests were applied. Tests including Kolmogorov-Smirnov, Shapiro-Wilk, and Levene were employed to identify differences between groups. One-way analysis of variance (ANOVA) was subsequently utilised for datasets with more than two groups that exhibited a normal distribution. Post-hoc tests, such as Tukey’s Honestly Significant Difference (HSD), were employed for pairwise comparisons. To indicate correlation coefficients for the cross tables created for categorical variables, Phi and Cramer’s V coefficients were used. In order to obtain the regression (prediction) equation of the independent variables and the dependent variable, a two-category logistic regression was performed since the dependent variable, tinnitus type (pathological and normal), is categorical data. Multiple logistic regression analysis (Backward Stepwise) method was used to show the combined effects of more than one independent variable on the dependent variable. Odds ratios (OR) were obtained. Statistical significance was determined using a margin of error of 0.05 and a confidence level of 0.95. A margin of error of 0.10 and a confidence level of 0.90 were applied in certain cases.

## Results

**Figure 1:**
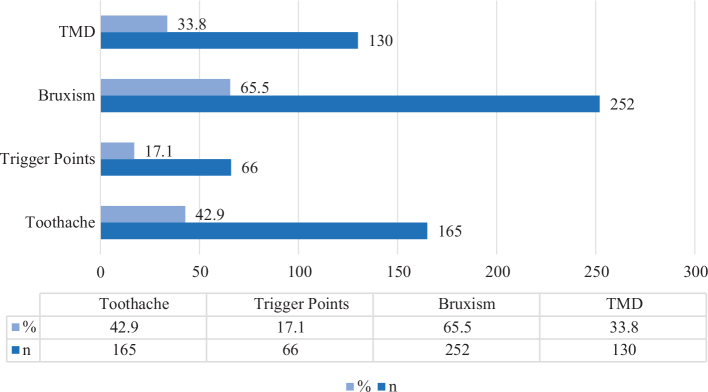
Distribution of oral maxillofacial diseases of patient with tinnitus.

Tinnitus was observed in 385 out of the 3,626 patients, resulting in a prevalence rate of 10.61%. The age range of the patients varied from 15 to 86 years, with a mean age of 42.66 ± 16.34 years. Predominantly, tinnitus was reported on the left side, accounting for 56.4% of cases. Of the patients, 38.4% were male, while 61.6% were female. The average age of male patients was 46.79 ± 17.20 years, whereas female patients had an average age of 40.08 ± 15.26 years. Concerning maxillofacial conditions, 66.2% of patients showed no evidence of TMD, with 19.5% having right-sided TMD and 14.3% presenting left-sided TMD. Bruxism was detected in 65.5% of the patients, while 34.5% did not exhibit this condition. Moreover, 82.9% of the patients had no trigger points, whereas 14.8% had trigger points on the right side and 12.3% on the left side. Toothache was reported by 42.9% of the patients, with 57.1% being free of toothache (Table 1). In general, the rate of patients with bruxism was higher followed by TMD and toothache, respectively. The lowest patient rate was seen in patients with presence trigger points (Figure 1). The type of tinnitus was categorised as normal in 47.8% of the patients and pathological in 52.2%. The patients predominantly described their tinnitus sounds as resembling ringing. Tinnitus was typically most commonly observed in patients for a duration of 2 to 5 years. The frequency of tinnitus was predominantly observed to be continuous. A tendency for tinnitus, accompanied by dental pain, was observed in 5.9% of the patients (Table 1).

**Table 1:** Demographic and clinical characteristics of patients with tinnitus.

Variables	Categories	*n*	%	Variables	Categories	*n*	%
Gender	Male	148	38.4	TMD	No	255	66.2
	Female	237	61.6		Right	75	19.5
Ear which has the tinnitus	Right	168	43.6		Left	55	14.3
	Left	217	56.4		Total	385	100.0
Type of tinnitus	Normal (<5 min )	184	47.8	Bruxism	No	133	34.5
	Pathological (>5 min)	201	52.2		Yes	252	65.5
Type of tinnitus sound	Buzz	76	19.7		Total	385	100.0
	Hum	103	26.8	Trigger points	No	319	82.9
	Whisper	4	1.0		Right	57	14.8
	Shrill	17	4.4		Left	9	2.3
	Ringing	185	48.1		Total	385	100.0
	Total	385	100.0	Toothache	No	220	57.1
Onset time of tinnitus	1 week – 1 month	18	4.7		Yes	165	42.9
	1 month – 1 year	38	9.9		Total	385	100.0
	2–5 years	126	32.7				
	5–10 years	52	13.5				
	10–20 years	43	11.2				
	Others	108	28.0				
The frequency of tinnitus	Continually	158	41.0				
	Several times a day	28	7.3				
	Several times a week	80	20.7				
	Several times a month	81	21.0				
	Several times a year	15	3.9				
	With toothache	23	5.9				
	**Mean ± Std. Deviation (Min-Max)**						
Age	42.66 ± 16.34 (15–86)						
Male	46.79 ± 17.20						
Female	40.08 ± 15.26						

The analysis revealed no statistically significant difference in mean age between patients with and without TMD. The mean age of patients without TMD findings was 43.68 ± 17.32, while those with TMD findings on the right side had a mean age of 39.72 ± 14.90, and those with left-sided TMD findings had a mean age of 41.94 ± 12.89; however, these differences were not statistically significant (*p* > 0.05). Statistically significant differences were observed in mean age between patients with and without bruxism (*p* = 0.000) and between patients with and without toothache (*p* = 0.001). Patients with bruxism had a mean age of 40.15 ± 15.84 years, whereas those without bruxism had a mean age of 47.41 ± 16.28 years. Similarly, patients with toothache had a mean age of 39.54 ± 15.69 years, whereas those without toothache had a mean age of 45.00 ± 16.46 years, and this difference was statistically significant (*p* = 0.001). There was a statistically significant difference in mean age between patients without trigger point, right and left, with a margin of error of 0.10 (*p* = 0.059). When multiple comparisons were made using the Tukey HSD test, the difference of 13.87 units in mean age between the right and left patients was statistically significant (*p* = 0.047). Patients with a trigger point on the left side were found to be older (Table 2).

**Table 2:** Association between age and maxillofacial diseases of patients with tinnitus.

	*N*	Mean	Std. Deviation	95% Confidence Interval for mean		Min	Max	*p*-value
				Lower bound	Upper bound			
TMD-None	255	43.68	17.32	41.55	45.82	15.00	86.00	0.171
TMD-Right	75	39.72	14.90	36.29	43.14	15.00	75.00	
TMD-Left	55	41.94	12.89	38.45	45.43	20.00	73.00	
Bruxism-None	133	47.41	16.28	44.61	50.20	17.00	78.00	0.000
Bruxism	252	40.15	15.84	38.19	42.12	15.00	86.00	
Trigger Points- None	319	42.73	16.37	40.92	44.53	15.00	86.00	0.059
Trigger Points-Right	57	40.45	15.86	36.24	44.66	18.00	75.00	
Trigger Points-Left	9	54.33	14.87	42.89	65.76	37.00	78.00	
Toothache-None	220	45.01	16.46	42.81	47.19	15.00	86.00	0.001
Toothache	165	39.54	15.69	37.13	41.95	15.00	74.00	
Total	385	42.66	16.34	41.02	44.30	15.00	86.00	

There was a 17.0% correlation between gender and TMD findings, which was statistically significant (*p* = 0.004; *χ*^2^ = 11.146). 75.0% of male had no TMD findings; 17.6% had TMD findings on the right side. 60.8% of female had no TMD; 20.7% had TMD on the right side. There was a 17.0% association between gender and bruxism, and this association was statistically significant (*p* = 0.004; *χ*^2^ = 6.843). Bruxism was present in 57.4% of male and 70.5% of female. There was no statistically significant difference between male and female in the presence of trigger points (*p* = 0.128; *χ*^2^ = 4.115). No trigger point was observed in 81.8% of male and 83.5% of female. The trigger point was observed on the right side in 17.6% of male and 13.1% of female. The association between gender and toothache was 13.4% and statistically significant (*p* = 0.009; *χ*^2^ = 6.923). While 34.5% of male reported toothache, 48.1% of female reported toothache (Table 3).

**Table 3:** Association between gender and maxillofacial diseases of patients with tinnitus.

		TMD				
		None *n* (%)	Yes *n* (%)			
			Right	Left	Total *n* (%)	*χ*^2^; *p*-value
Gender	Male	111 (75)	26 (17.6)	11 (7.4)	148 (100)	11.146; 0.004 Cramer’s *V* = 0.170
	Female	144 (60.8)	49 (20.7)	44 (18.6)	237 (100)	
Total		255 (66.2)	75 (19.5)	55 (14.3)	385 (100)	
		Bruxism				
		None *n* (%)	Yes *n* (%)		Total *n* (%)	*χ*^2^; *p*-value
Gender	Male	63 (42.6)	85 (57.4)		148 (100)	6.843; 0.009 Phi = 0.170
	Female	70 (29.5)	167 (70.5)		237 (100)	
Total		133 (34.5)	252 (65.5)		385 (100)	
		Trigger Points				
		None *n* (%)	Yes *n* (%)			
	Right	Left	Total *n* (%)	*χ*^2^; *p*-value
Gender	Male	121 (81.8)	26 (17.6)	1 (0.7)	148 (100)	4.115; 0.128 Cramer’s *V* = 0.103
	Female	198 (83.5)	31 (13.1)	8 (3.4)	237 (100)	
Total		319 (82.9)	57 (14.8)	9 (2.3)	385 (100)	
		Toothache				
		None *n* (%)	Yes *n* (%)		Total *n* (%)	*χ*^2^; *p*-value
Gender	Male	97 (65.5)	51 (34.5)		148 (100)	6.923; 0.009 Phi = 0.134
	Female	123 (51.9)	114 (48.1)		237 (100)	
Total		220 (57.1)	165 (42.9)		385 (100)	

**Figure 2:**
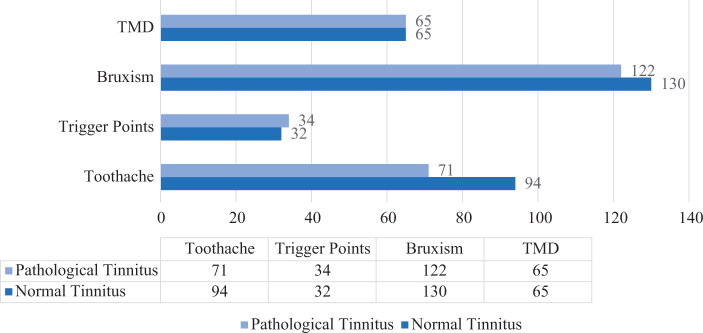
Distribution of oral and maxillofacial diseases by type of tinnitus.

No statistically significant associations were found between the type of tinnitus and TMD findings or trigger points (*p* > 0.05). However, a statistically significant association was observed between the type of tinnitus and bruxism (*p* = 0.040). Among those with normal tinnitus type, 70.7% had bruxism and 29.3% had no bruxism. Among those with pathological tinnitus type, 60.7% had bruxism and 39.3% did not. Furthermore, a statistically significant association was found between the type of tinnitus and toothache (*p* = 0.002). Among those with normal tinnitus type, 51.1% had toothache and 48.9% did not. Among those with pathological tinnitus, 35.3% had toothache and 64.7% did not (Figure 2). There was a 19.3% correlation between which ear the tinnitus was in and the presence of TMD, which was statistically significant (*p* = 0.001; *χ*^2^ = 14.307). No TMD was found in 65.5% of the patients with right ear tinnitus and 66.8% with left ear tinnitus. In 26.2% of the patients with tinnitus in the right ear and 14.3% with tinnitus in the left ear, the TMD finding was on the right side. In 8.3% of the patients with tinnitus in the right ear and 18.9% with tinnitus in the left ear, the TMD was located on the left side. There was a 17.5% correlation between which ear the tinnitus was in and the presence of a trigger point, which was statistically significant (*p* = 0.003; *χ*^2^ = 11.848). In 20.8% of the patients with tinnitus in the right ear and 10.1% of those with tinnitus in the left ear, the points were on the right side. In 0.6% of the patients with tinnitus in the right ear and 3.7% with tinnitus in the left ear, the trigger points were on the left side.

There was no statistically significant relationship between tinnitus duration and the presence of TMD, bruxism, trigger point (*p* = 0.521, *χ*^2^ = 70.721; *p* = 0.202, *χ*^2^ = 42.798; *p* = 0.996, *χ*^2^ = 44.421). There was a 37.0% correlation between tinnitus duration and toothache which was statistically significant (*p* = 0.035; *χ*^2^ = 52.835). There was a 38.7% correlation between tinnitus frequency and toothache which was statistically significant (*p* = 0.000; *χ*^2^ = 57.627). There was no statistically significant relationship between the frequency of tinnitus and the presence of TMD and bruxism (*p* = 0.815; *χ*^2^ = 30.126) (*p* = 0.800; *χ*^2^ = 13.711). There was a 36.8% relationship between the frequency of tinnitus and the presence of trigger points, which was statistically significant (*p* = 0.000; *χ*^2^ = 104.522).

In addition, the effects of TMD, bruxism, toothache and trigger points variables on tinnitus type were evaluated together. The effects of bruxism and toothache on the type of tinnitus were found to be statistically significant. The presence of bruxism increased the risk of pathological tinnitus type by 1.456 fold (95% Confidence Interval [CI]: 0.945–2.442). The presence of toothache increased the risk of pathological tinnitus type by 1.839 fold (95% CI: 1.218–2.778). The presence of toothache affected the risk of tinnitus being pathological more than the presence of bruxism (Table 4).

**Table 4:** Evaluation of the effect oral and maxillofacial disease on tinnitus type by multiple logistic regression analysis.

								95% CI for OR	
	Variables	B	SE	Wald	df	*p*-value	OR	Lower	Upper
**Step 1^a^**	TMD (No)			0.72	2	0.695			
	TMD (Right)	0.05	0.31	0.02	1	0.871	1.051	0.578	1.910
	TMD (Left)	−0.17	0.36	0.24	1	0.622	0.836	0.411	1.701
	Bruxism (Yes)	0.38	0.22	3.06	1	0.080*	1.474	0.954	2.275
	Toothache (Yes)	0.61	0.21	8.22	1	0.004**	1.830	1.211	2.766
	Trigger Points (No)			0.15	2	0.926			
	Trigger Points (Right)	0.27	0.69	0.15	1	0.694	1.315	0.336	5.147
	Trigger Points (Left)	0.26	0.73	0.13	1	0.717	1.307	0.308	5.540
	Constant	−0.65	0.73	0.81	1	0.369	0.521		
**Step 2^b^**	TMD (No)			0.75	2	0.687			
	TMD (Right)	0.06	0.30	0.03	1	0.843	1.062	0.586	1.924
	TMD (Left)	−0.17	0.36	0.22	1	0.635	0.842	0.415	1.709
	Bruxism (Yes)	0.38	0.22	3.02	1	0.082*	1.469	0.952	2.266
	Toothache (Yes)	0.61	0.21	8.33	1	0.004**	1.837	1.216	2.775
	Constant	−0.39	0.31	1.63	1	0.201	0.674		
**Step 3** ^c^	Bruxism (Yes)	0.37	0.22	2.91	1	0.088*	1.456	0.945	2.242
	Toothache (Yes)	0.61	0.21	8.39	1	0.004**	1.839	1.218	2.778
	Constant	−0.38	0.17	5.18	1	0.023	0.679		

B: Beta; SE: Standard Error; df: Degrees of Freedom; CI: Confidence Interval; OR: Odds Ratio.

a Variable(s) entered on step 1: TMD, Bruxism, Toothache, Trigger Points.

b Variable(s) removed on step 2: Trigger Points.

c Variable(s) removed on step 3: TMD.

* :*p* < 0.10,

** :*p* < 0.05.

## Discussion

Tinnitus is a common symptom that significantly impacts individuals’ overall quality of life [26]. Despite the association of bruxism and syndromes with tinnitus originating from TMD, there is no model explaining how tinnitus occurs in relation to dental occlusion and TMD [20, 24, 27]. This study aims to assess the prevalence of tinnitus and its potential association with maxillofacial diseases in patients who routinely seek care at a faculty of dentistry. To the best of our knowledge, this is the first study to investigate the prevalence of tinnitus among patients attending a faculty of dentistry.

The results of this study indicate that the prevalence of tinnitus among patients presenting to the dental faculty is 10.61%. A recent systematic review reports that tinnitus affects approximately 14.0% of adults [6]. This finding has yielded results closely aligned with this study. The incidence of tinnitus increases with age [28]. In this study, the mean age of the patients was 42.66 ± 16.34. There is no consensus in the literature regarding the relationship between gender and tinnitus [6]. While it has been reported that exposure to noise is higher in male compared to female, leading to a higher prevalence of tinnitus in male, another study argues that there is no relationship between gender and tinnitus [29, 30]. In this study, the proportion of female with tinnitus was higher than male. However, this could be attributed to the higher prevalence of TMD in female in this study, as it has been found that the prevalence of tinnitus is higher in female with TMD compared to men [31–33]. These findings in female may be associated with stress [34]. Tinnitus can be perceived in one or both ears [35]. However, it has also been reported to be more common in one ear [36]. Axelsson et al. reported a higher prevalence of tinnitus in the left ear in their study. The reason for this phenomenon remains unclear [37]. In this study, similar to the findings of Axelsson et al., tinnitus was more frequently observed in the left side [37]. In this study, most patients described the sound they heard as ringing, and patients often had pathological tinnitus.

There are anatomical and functional connections within the medulla oblongata that links the trigeminal and dorsal column systems of the somatosensory system with the cochlear nucleus of the auditory system [38]. Additionally, the activity of the dorsal cochlear nucleus can be influenced by stimulation of non-auditory structures. Consequently, tinnitus may arise from the activation of underlying autonomic interactions [39]. These considerations shed light on the potential relationship between maxillofacial diseases and tinnitus. Notably, maxillofacial diseases are considered potential risk factors for tinnitus development and could impact the severity of its symptoms [7]. Reports indicate that it may occur subsequent to maxillary fractures, orthognathic surgery, and maxillary sinus floor elevation procedure [40–42]. Temporomandibular disorder, herniation of Foramen Huschke, myofascial pain in the head/neck region, presence of bruxism, and toothache have also been suggested as potential contributors to tinnitus symptoms [9, 15, 43, 44]. In patients with TMD and tinnitus, appropriate TMD treatment can reduce the severity of tinnitus [31, 45]. Botulinum toxin, a locally applicable neurotoxin that inhibits acetylcholine release at the neuromuscular junction, is used as a beneficial clinical alternative for the treatment of head and neck pain, and functional disorders. Particularly in cases where the problem is presumed to be primarily muscle-related and resistant myofascial pain related to TMD, it is utilised as an adjunct to existing conservative treatments, targeting the hypertrophy of the masseter muscle [46–49]. Additionally, it is commonly employed for musculoskeletal pathology-related tinnitus, resulting from tremors in the soft palate, stapedius myoclonus, and contractions in the masseter and lateral pterygoid muscles [50]. In this study, among the maxillofacial symptoms associated with tinnitus, bruxism was the most common, followed by toothache, TMD, and the presence of trigger points in the masticatory muscles. Interestingly, toothache was more prevalent in patients experiencing short-term tinnitus. Additionally, it was found that the presence of bruxism and toothache increased the risk of pathological tinnitus in patients.

Temporomandibular disorder is a risk factor for the development of tinnitus [51]. Tinnitus is commonly observed in patients with TMD [52]. The prevalence of tinnitus in patients with TMD may vary widely, ranging from 3.7% to 70.0% [43]. In a study conducted by Çebi et al., the prevalence of tinnitus was found to be 11.46% in patients presenting with TMD complaints [53]^.^ Lam et al., in their study involving patients with tinnitus, reported that 64.0% of the patients had TMD [18]. In this study, TMD was found in 33.8% of patients with tinnitus. The number of TMD patients with tinnitus was higher in females compared to males, although it showed no correlation with age. The association between tinnitus and TMD is primarily ipsilateral [31]. This suggests that neural interactions between TMD and tinnitus may occur, potentially due to nerves’ sensitivity to ipsilateral stimuli [54]. In the current study, a significant relationship was established between the side of the tinnitus symptom and the side of the TMD symptom, with the left side being more frequently affected. However, the type, frequency, and time of tinnitus were not associated with TMD.

The masticatory, palatal, and tympanic muscles share a common phylogenetic origin and are innervated by the trigeminal nerve. Due to this shared innervation, abnormal usage of the masticatory system can lead to the contraction of these muscles. Consequently, otological symptoms may arise as a result of increased tension in the tympanic membrane [14]. Increased tension in the jaw muscles has been considered a risk factor for tinnitus [12]. It has been reported that muscle tension is common in patients with tinnitus and may be due to functional disorders of the jaw muscles [55]. In a study by Fernandes et al., a prevalence of tinnitus exceeding 50% was observed in patients with bruxism, and it was reported that TMD in combination with bruxism is associated with a higher degree of comorbidity with tinnitus compared to TMD alone [56]. Peroz concluded in his study that tinnitus is associated with pathologies of the jaw muscles, with occlusal instability and bruxism being the likely causes [55]. Camparis et al. reported in their study that the frequency of tinnitus is significantly higher in patients with sleep bruxism [9]. In this study, bruxism was observed in 65% of patients with tinnitus and presence of bruxism was more common in female than in male. The ages of patients with bruxism were significantly younger. Interestingly, bruxism was more prevalent among patients experiencing normal tinnitus. However, no association was found between the type and duration of tinnitus.

Tinnitus can be influenced or modulated by inputs originating from the somatosensory and somatomotor systems. Therefore, sensory or motor stimuli, such as muscle contractions, mechanical pressure applied to myofascial trigger points, or transcutaneous electrical stimulation, can alter the intensity or severity of tinnitus [57]. Transient modulation of tinnitus may occur when digital pressure is applied to trigger points [58]. In patients with TMD reporting tinnitus, the prevalence of myofascial pain is higher compared to TMD patients without tinnitus [59]. In a study conducted by Ravuri, it was reported that patients with myofascial pain have a higher incidence of tinnitus compared to other forms of TMD [60]. In this study, the presence of trigger points in masticatory muscles among patients with tinnitus was observed in 27.1%. No relationship was found between trigger points and the type and duration of tinnitus. However, a significant association was observed between the side of tinnitus and the side with trigger points.

Tinnitus has a multifactorial aetiology similar to pain, and addressing or eliminating one of the contributing factors may lead to complete resolution of symptoms [61]. Individuals experiencing toothache are more likely to have tinnitus compared to those without such symptoms [15]. Additionally, the prevalence of tinnitus may be higher in patients presenting with both TMD and toothache compared to those with TMD alone [15]. In this study, prevalence of toothache in patients with tinnitus was 42.9%. This higher rate may be attributed to toothache being one of the most common reasons for patients to seek dental care. Toothache was more prevalent in males compared to females, and the patients were significantly younger. However, the correlation between the frequency of tinnitus and toothache, along with the higher prevalence of normal tinnitus in patients with toothache, may indicate mutual influences between acute pathologies of the auditory and masticatory systems. Additionally, interestingly it was also found that tinnitus was triggered by toothache in some of the patients in the study. The fate of the tinnitus symptom following the treatment of toothache is a topic of interest.

This study has several limitations that should be taken into consideration. Firstly, the evaluation of tinnitus relied on subjective reports obtained from patients, which may introduce some degree of subjectivity and variability. One of the limitations of the study is the absence of age restrictions, as well as the lack of categorisation for tooth deficiency and dental caries. Additionally, the lack of objective methods for evaluating bruxism, reliance on clinical examination for occlusion assessment, absence of patient follow-up, and inability to assess treatment outcomes are also limitations. To gain a more comprehensive understanding of tinnitus associated with oral and maxillofacial diseases, future research should aim for longer follow-up periods and involve larger and more diverse populations.

In conclusion, there is an association between maxillofacial diseases and tinnitus. Assessment of these diseases should become routine in the management of tinnitus. According to the results of this study, subjective tinnitus was observed in conjunction with bruxism, toothache, TMD, and the presence of trigger points, respectively. Particularly, bruxism and toothache have the potential to increase pathological tinnitus. A multidisciplinary approach to the symptom of tinnitus may improve patients’ quality of life and expedite treatment.
